# Adhesion molecules in focus: mechanistic pathways and therapeutic avenues in sickle cell vaso-occlusion – a narrative review

**DOI:** 10.1097/MS9.0000000000003619

**Published:** 2025-07-18

**Authors:** Emmanuel Ifeanyi Obeagu

**Affiliations:** a Department of Biomedical and Laboratory Science, Africa University, Mutare, Zimbabwe

**Keywords:** adhesion molecules, endothelial dysfunction, inflammation, sickle cell disease, vaso-occlusion

## Abstract

Sickle cell disease (SCD) is a genetic disorder characterized by the presence of sickle-shaped red blood cells (sRBCs), which are prone to occluding small blood vessels, leading to severe pain and organ damage. One of the critical mechanisms driving vaso-occlusion in SCD is the interaction between sRBCs, leukocytes, platelets, and endothelial cells, mediated by adhesion molecules. These molecules, including intercellular adhesion molecule-1, vascular cell adhesion molecule-1, selectins, and integrins, play a significant role in promoting the adhesion of these cells to the vascular endothelium, exacerbating inflammation, and contributing to the obstruction of blood flow. Understanding how these adhesion molecules participate in the pathophysiology of vaso-occlusion offers valuable insights into potential therapeutic strategies to mitigate the impact of this debilitating condition. The role of adhesion molecules in SCD-induced vaso-occlusion has been well-documented, with multiple studies showing that their upregulation enhances the interaction between sickled and non-sickled cells and the endothelium. This interaction initiates a cascade of inflammatory responses that worsen microvascular occlusion, leading to tissue ischemia and chronic complications. The expression of adhesion molecules such as E-selectin, P-selectin, and integrins on both the endothelial surface and the sickle cell membrane is critical for the progression of these vaso-occlusive events. Inflammation-induced overexpression of these molecules increases cell adhesion, exacerbating the frequency and severity of vaso-occlusive crises and contributing to long-term organ damage in SCD patients.

## Introduction

Sickle cell disease (SCD) is a genetic hemoglobinopathy primarily affecting individuals of African, Mediterranean, and Middle Eastern descent. It is caused by a mutation in the gene encoding hemoglobin, resulting in the production of hemoglobin S (HbS). Under low oxygen conditions, HbS causes red blood cells (RBCs) to adopt a rigid, sickle-shaped morphology, which reduces their ability to deform as they travel through microvasculature. This leads to blood flow obstruction, known as vaso-occlusion, which is responsible for much of the morbidity and mortality associated with the disease. The clinical manifestations of vaso-occlusion are diverse, ranging from acute painful crises to chronic organ damage, including stroke, pulmonary hypertension, and kidney dysfunction^[1,2]^. The process of vaso-occlusion in SCD is multifactorial, with interactions between sickled RBCs, leukocytes, platelets, and endothelial cells playing a central role. Adhesion molecules, which are cell surface proteins involved in the binding of cells to one another or the extracellular matrix, are crucial mediators of these interactions. These molecules facilitate the adhesion and aggregation of sickle cells, platelets, and white blood cells to the endothelial lining of blood vessels, further promoting the formation of microvascular occlusions. The upregulation of adhesion molecules in response to inflammation exacerbates these processes, perpetuating the cycle of vaso-occlusion^[3,4]^. Among the adhesion molecules involved in SCD, intercellular adhesion molecule-1 (ICAM-1), vascular cell adhesion molecule-1 (VCAM-1), selectins (such as E-selectin and P-selectin), and integrins are all of particular importance. ICAM-1 and VCAM-1 are expressed on the surface of endothelial cells and play a key role in the adhesion of leukocytes and RBCs to the vessel wall. Selectins, which are involved in the initial rolling and attachment of cells to the endothelium, contribute to the recruitment of both neutrophils and sickle-shaped red blood cells (sRBCs) to sites of occlusion. Integrins mediate the firm adhesion of cells to the endothelial surface, facilitating their accumulation at sites of injury or inflammation. In SCD, these adhesion molecules are often overexpressed due to the chronic inflammatory state induced by sickled cells and their interaction with the endothelial cells^[5,6]^.

sRBCs themselves also play a crucial role in promoting the expression of adhesion molecules. The rigidity and altered surface properties of sickled cells increase their interactions with the vascular endothelium. In addition, sickled RBCs can directly activate endothelial cells and promote the release of inflammatory cytokines and adhesion molecules, further amplifying the inflammatory response. The interaction between sickle cells and the endothelium leads to the formation of a “sticky” vascular surface, where RBCs, leukocytes, and platelets can more readily adhere, thus increasing the risk of vaso-occlusion[7]. Vaso-occlusion is not solely the result of physical blockage of blood vessels by sickle cells. The inflammatory response elicited by the adhesion of cells to the endothelium also plays a significant role in amplifying the severity of vaso-occlusive events. Once adhered to the vessel wall, leukocytes release pro-inflammatory mediators that further activate the endothelial cells and recruit additional inflammatory cells to the site. This inflammatory cascade exacerbates endothelial dysfunction and further impairs blood flow, resulting in a vicious cycle of inflammation and occlusion[8]. In addition to the primary vascular effects, vaso-occlusion in SCD has long-term implications for organ function. Chronic ischemia caused by repeated vaso-occlusive events leads to progressive organ damage, including infarction of the spleen, kidneys, lungs, and brain. The cumulative impact of vaso-occlusion on these organs contributes significantly to the morbidity and mortality associated with SCD. Furthermore, the debilitating nature of recurrent pain crises and organ dysfunction has a profound impact on the quality of life for individuals with SCD, leading to substantial healthcare burden and reduced life expectancy[9]. Therapeutic strategies aimed at mitigating vaso-occlusion in SCD have traditionally focused on managing symptoms and preventing the complications of the disease. Blood transfusions, hydroxyurea, and pain management are commonly employed to reduce the frequency and severity of vaso-occlusive crises. However, these treatments are often not fully effective in preventing the underlying cellular interactions that cause vaso-occlusion. Recent advancements have led to the exploration of more targeted therapies that aim to disrupt the molecular pathways involved in the adhesion of sickle cells and leukocytes to the endothelium[1]. Targeting adhesion molecules presents an exciting avenue for the development of novel therapeutic strategies to treat vaso-occlusion in SCD. By blocking the interaction of sickle cells and inflammatory cells with the endothelial surface, adhesion molecule inhibitors could prevent or reduce the occurrence of vaso-occlusive crises. Additionally, gene-based therapies, which aim to modify the expression of adhesion molecules or alter the function of sickle hemoglobin, hold the potential for a more durable solution to mitigate the devastating effects of this disease[10].

## Aim

The aim of this review is to explore the various molecular and cellular pathways contributing to vaso-occlusion in SCD and to discuss the current and emerging therapeutic strategies targeting these pathways.

### Justification of the review

Vaso-occlusion is a hallmark and one of the most debilitating manifestations of SCD, leading to acute pain crises, organ damage, and a diminished quality of life. The complexity of its pathophysiology involves multiple cellular interactions, including the adhesion of sRBCs, leukocytes, platelets, and endothelial cells, all of which contribute to the obstruction of microvasculature. Despite ongoing advances in the understanding of SCD, current therapeutic options primarily focus on alleviating symptoms rather than targeting the root cause of vaso-occlusion. This gap underscores the need for a comprehensive review that consolidates the latest insights into the molecular mechanisms of vaso-occlusion and explores novel therapeutic approaches aimed at addressing these pathways. This review is timely and necessary given the growing interest in adhesion molecules as key drivers of vaso-occlusion. Targeting these molecules, such as selectins, integrins, and VCAM-1, has shown promising results in preclinical studies and early-phase clinical trials. However, there remains a lack of in-depth synthesis of existing data and an understanding of how these therapies could be effectively integrated into clinical practice. By critically evaluating the current research on adhesion molecule-targeted therapies, RBC deformability modulation, anti-inflammatory strategies, and gene therapies, this review aims to provide a thorough examination of the various pathways to mitigate vaso-occlusion in SCD. Furthermore, with new treatment options such as gene therapy and combination regimens being developed, there is a pressing need to guide future research in optimizing these therapies and addressing their limitations. This review seeks to identify gaps in current research, propose future directions for exploring therapeutic interventions, and ultimately support the development of more effective and targeted treatments for SCD patients. These findings will not only enhance the understanding of the molecular drivers of vaso-occlusion but also contribute to improving the clinical outcomes and quality of life for individuals living with SCD^[11–15]^.

**HIGHLIGHTS**

- Adhesion molecules are key contributors to vaso-occlusion in sickle cell disease.
- Targeting adhesion molecules can reduce microvascular blockage and pain crises.
- Selectins, integrins, and VCAM-1 play pivotal roles in pathophysiology.
- Therapeutic strategies focus on modulating adhesion molecule activity.
- Innovative treatments like gene therapy show promise for long-term management.

## Review methodology

### Literature search strategy

A thorough and systematic literature search was conducted to gather relevant articles. Databases such as PubMed, Scopus, Google Scholar, and Web of Science were used to identify peer-reviewed journal articles, clinical trials, review articles, and book chapters. The following keywords and search terms were employed: “sickle cell disease,” “vaso-occlusion,” “adhesion molecules,” “selectins,” “integrins,” “VCAM-1,” “therapeutic strategies,” “gene therapy,” “inflammation,” “cell adhesion,” “red blood cell deformability,” and “platelet inhibition.” The inclusion criteria focused on articles that explored the pathophysiology of vaso-occlusion, the role of adhesion molecules, and therapeutic interventions targeting these pathways.

### Study selection and inclusion criteria

Studies included in the review were selected based on the following criteria:

- Research focusing on the pathophysiology of vaso-occlusion in SCD, including the role of adhesion molecules, RBC deformability, inflammation, and endothelial cell interactions.
- Studies evaluating therapeutic strategies aimed at reducing vaso-occlusion, including pharmacological treatments (e.g., hydroxyurea, anti-selectin therapies), gene therapy, blood transfusions, and anti-inflammatory approaches.
- Articles that provided experimental data, clinical trial results, or in-depth reviews of therapeutic strategies.

Exclusion criteria included studies that:

Did not focus specifically on SCD or vaso-occlusion.

Were non-English language articles, as translation was not feasible.

Focused on basic science with no direct relevance to therapeutic interventions or clinical outcomes.

### Adhesion molecules and vaso-occlusion in SCD

Vaso-occlusion is the hallmark pathological event in SCD, leading to painful crises and organ damage. This process is primarily driven by the interaction of sRBCs, leukocytes, platelets, and endothelial cells, all of which are influenced by adhesion molecules. These molecules, which include selectins, integrins, and immunoglobulin superfamily members such as ICAM-1 and VCAM-1, are crucial for the adhesion of these cells to the vascular endothelium, where they initiate and propagate microvascular occlusions. Understanding how these adhesion molecules contribute to vaso-occlusion is key to identifying therapeutic targets that can mitigate the severe complications of SCD^[16,17]^. The role of adhesion molecules in SCD-induced vaso-occlusion is multifaceted. sRBCs exhibit increased adhesive properties due to alterations in their surface structure, including the expression of adhesion receptors and the exposure of phosphatidylserine, which make them more prone to sticking to the endothelium. In addition to this, sickled cells can directly activate endothelial cells through the release of inflammatory cytokines and cellular components such as heme, leading to the upregulation of adhesion molecules. This creates a feedback loop that enhances the adhesion of sickle cells and leukocytes to the endothelial surface, promoting occlusion. Key adhesion molecules, such as E-selectin and P-selectin, are involved in the initial tethering and rolling of sickle cells and leukocytes, while integrins and ICAM-1/VCAM-1 mediate firm adhesion and transmigration^[18–20]^.

One of the most studied adhesion molecules in SCD is E-selectin, a cell adhesion protein expressed by endothelial cells in response to inflammation. E-selectin facilitates the early interaction between leukocytes and endothelial cells, a critical step in the recruitment of immune cells to sites of vaso-occlusion. In the context of SCD, this molecule plays an important role in promoting the adhesion of sickle cells, further exacerbating the occlusion. Similarly, P-selectin, which is stored in platelet granules and endothelial cells, is rapidly mobilized during vaso-occlusive events to facilitate platelet-leukocyte aggregation and enhance the formation of occlusions. Other adhesion molecules like ICAM-1 and VCAM-1 are upregulated in the inflamed endothelium and mediate the firm attachment of leukocytes to endothelial cells, promoting leukocyte trafficking into inflamed tissues and contributing to the exacerbation of vaso-occlusion^[21,22]^. Integrins, another class of adhesion molecules, are essential for the firm adhesion of cells to the endothelium. Integrins, such as α4β1 (VLA-4), are particularly involved in the adhesion of sickle cells and leukocytes to VCAM-1 expressed on the endothelial surface. This interaction not only contributes to the arrest of blood flow in small vessels but also facilitates the inflammatory response by recruiting more immune cells. The engagement of integrins with their ligands, including VCAM-1 and fibronectin, is critical for the establishment and maintenance of microvascular occlusions in SCD, thereby promoting tissue ischemia and further damage to organs[23].

**Figure 1.**
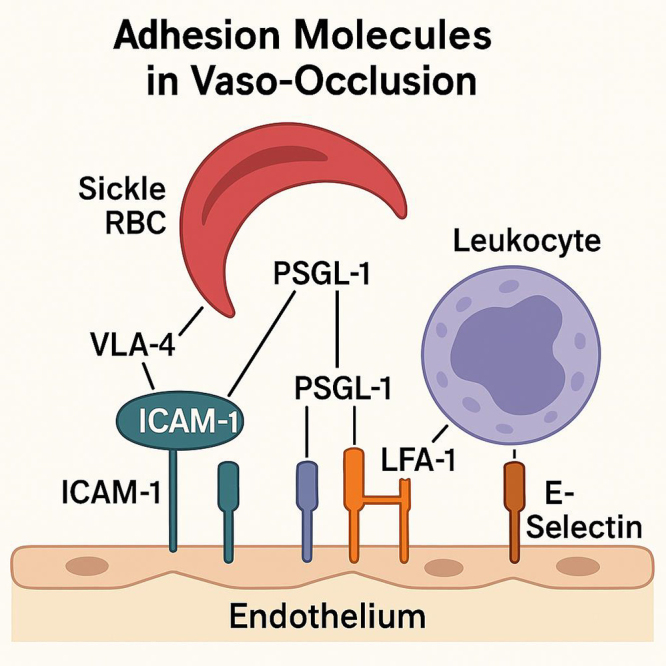
Adhesion molecule interactions in vaso-occlusion.

In addition to the involvement of adhesion molecules on the endothelial surface, sRBCs themselves are significant contributors to the process of vaso-occlusion. The abnormal shape and rigid properties of sickle cells make them more likely to adhere to the endothelium, and this adhesion is enhanced by the expression of adhesion molecules such as CD36 and α4β1 integrins. These molecules facilitate interactions with endothelial adhesion proteins, such as VCAM-1, promoting cell clustering and the formation of microvascular plugs. Furthermore, sickled cells release inflammatory mediators that activate endothelial cells, creating a pro-adhesive environment that exacerbates the vicious cycle of adhesion and occlusion^[24,25]^. The upregulation of adhesion molecules also leads to the recruitment of neutrophils, which are central to the pathophysiology of vaso-occlusion. Neutrophils, when activated, interact with endothelial adhesion molecules such as ICAM-1 and VCAM-1, which in turn accelerates the formation of microthrombi and exacerbates endothelial dysfunction (Fig. 1). These interactions are often linked to the production of reactive oxygen species (ROS) and the release of cytokines, further contributing to the inflammatory environment that worsens occlusion. The role of neutrophils in SCD highlights the complex interplay between immune cells and adhesion molecules in driving the pathology of vaso-occlusion^[26,27]^. Table 1 shows adhesion molecules and their roles in vaso-occlusion in SCD.

**Table 1:** Adhesion molecules and their roles in vaso-occlusion in sickle cell disease

Adhesion molecule	Cellular source	Ligand/binding partner	Role in vaso-occlusion	Clinical significance
**CAM-1**	Endothelial Cells	VLA-4 (α4β1 integrin) on RBCs and leukocytes	Mediates firm adhesion of sickled RBCs and leukocytes to endothelium	Elevated in VOC; potential therapeutic target
**ICAM-1**	Endothelial Cells	LFA-1 (αLβ2 integrin) on leukocytes	Promotes leukocyte-endothelium adhesion and transmigration	Contributes to inflammation and leukocyte plugging
**P-Selectin**	Endothelial Cells, Platelets	PSGL-1 on leukocytes and RBCs	Facilitates initial rolling and tethering of cells on endothelium	Key driver of VOC initiation; target of crizanlizumab therapy
**E-Selectin**	Endothelial Cells	ESL-1, CD44, and PSGL-1 on leukocytes	Mediates leukocyte rolling and activation	Involved in leukocyte recruitment and vascular occlusion
**L-Selectin**	Leukocytes	Endothelial glycoproteins	Involved in leukocyte homing and migration	Less directly involved in VOC but contributes to inflammation
**VLA-4 (α4β1)**	Reticulocytes, Leukocytes	VCAM-1 on endothelial cells	Promotes firm adhesion of reticulocytes and leukocytes to endothelium	Elevated in SCD; promotes cell clustering and obstruction
**LFA-1 (αLβ2)**	Neutrophils, T cells	ICAM-1 on endothelium	Supports leukocyte adhesion and transmigration	Enhances inflammatory cell accumulation
**CD36**	RBCs, Monocytes	Thrombospondin, endothelial ligands	Mediates sickled RBC and monocyte adhesion to endothelium and extracellular matrix	Correlates with disease severity and frequency of VOC
**αvβ3 Integrin**	Endothelial Cells	Vitronectin, fibronectin	Facilitates endothelial cell adhesion and angiogenesis	Implicated in endothelial activation and remodeling
**PSGL-1**	Leukocytes, RBCs	P-Selectin, E-Selectin	Crucial for initiating rolling on activated endothelium	Plays a central role in leukocyte recruitment and adhesion

### Pathophysiology of vaso-occlusion in SCD

Vaso-occlusion is a central pathological event in SCD that leads to the hallmark complications such as pain crises, organ damage, and reduced lifespan. It occurs when sRBCs block blood flow through small blood vessels, obstructing the delivery of oxygen and nutrients to tissues. The underlying mechanism of vaso-occlusion in SCD is multifactorial, involving complex interactions between sRBCs, white blood cells (leukocytes), platelets, endothelial cells, and various adhesion molecules. These interactions promote inflammation, endothelial dysfunction, and microvascular obstruction, resulting in ischemia, pain, and tissue injury^[28,29]^. At the molecular level, vaso-occlusion begins when HbS, the abnormal form of hemoglobin in individuals with SCD, polymerizes upon deoxygenation, causing RBCs to adopt a rigid, crescent shape. This abnormal morphology impairs the deformability of RBCs, making it difficult for them to pass through narrow capillaries. These sickle-shaped cells become more prone to sticking to the endothelial lining of blood vessels, leading to the formation of blood clumps and the aggregation of other blood components, such as leukocytes and platelets. As the sickled cells accumulate and adhere to the vessel wall, they form a microvascular plug that obstructs blood flow[30].

Adhesion molecules play a crucial role in the development of vaso-occlusion by facilitating the interaction between sRBCs, platelets, leukocytes, and the endothelial cells lining blood vessels. These molecules, such as selectins, integrins, and immunoglobulin superfamily members, such as ICAM-1 and VCAM-1, are upregulated in response to the inflammation caused by sickling. This upregulation leads to the recruitment and adhesion of additional sickled cells, white blood cells, and platelets to the site of occlusion, further exacerbating the obstruction and creating a vicious cycle of cellular accumulation. E-selectin and P-selectin, for instance, mediate the initial tethering and rolling of cells on the endothelial surface, while integrins and VCAM-1 mediate the firm adhesion and transmigration of cells, contributing to the progression of vaso-occlusion^[31,32]^. The activation of leukocytes, particularly neutrophils, further amplifies the pathophysiological process. Neutrophils, upon activation, adhere to the endothelial cells via ICAM-1 and VCAM-1, and release pro-inflammatory cytokines and ROS, which further damage the endothelial cells. This inflammatory response not only enhances the adhesion of sickled cells but also promotes the formation of microthrombi, exacerbating the obstruction of blood flow. The continued inflammatory signaling from activated leukocytes and platelets results in endothelial dysfunction, which impairs the ability of blood vessels to dilate and respond to shear stress, increasing the likelihood of vascular occlusion^[33,34]^.

In addition to the mechanical blockage of blood vessels, vaso-occlusion triggers a cascade of biochemical events that lead to further tissue damage. Ischemia resulting from obstructed blood flow causes cellular hypoxia, which in turn activates a variety of stress responses. Hypoxia-inducible factors are upregulated, promoting the expression of adhesion molecules and inflammatory cytokines, which exacerbate the inflammatory environment and contribute to endothelial injury. Furthermore, the accumulation of metabolites, such as lactate, and the release of cellular debris from damaged tissues can amplify the inflammatory response, further perpetuating the cycle of vaso-occlusion and tissue damage^[35,36]^. Over time, repeated vaso-occlusive events contribute to chronic tissue ischemia and organ damage. Organs such as the spleen, kidneys, lungs, and brain are particularly vulnerable to the effects of vaso-occlusion. The progressive ischemia and infarction of these organs result in significant long-term complications, including stroke, pulmonary hypertension, renal failure, and splenic dysfunction. In the bone marrow, chronic vaso-occlusion can lead to the development of osteonecrosis. Additionally, the recurring ischemia and inflammation can accelerate endothelial dysfunction, contributing to the development of atherosclerosis in larger blood vessels^[37,38]^.

In the bone marrow, the chronic inflammatory environment induced by vaso-occlusion affects hematopoiesis, further complicating the pathophysiology of the disease. The persistence of low-grade inflammation and oxidative stress in SCD patients leads to alterations in the bone marrow microenvironment, which can result in altered RBC production and exacerbated hemolysis. The increased turnover of sickle cells releases free hemoglobin, heme, and other pro-inflammatory substances into circulation, further contributing to oxidative stress, endothelial injury, and vascular dysfunction^[39,40]^. Vaso-occlusion in SCD is not only a consequence of mechanical blockage but is intricately linked to a chronic inflammatory state that amplifies the disease’s pathophysiology. The inflammatory environment, sustained by the repeated interactions of sRBCs, white blood cells, platelets, and endothelial cells, underpins the vicious cycle of vaso-occlusion. Addressing this inflammation through targeted therapies aimed at adhesion molecules, inflammatory cytokines, or oxidative stress pathways holds promise for reducing the severity of vaso-occlusion and mitigating the long-term consequences of SCD. However, challenges remain in translating these insights into effective clinical treatments, necessitating ongoing research into the molecular mechanisms and therapeutic options for SCD^[41,42]^.

### The significance of vaso-occlusion in SCD

Vaso-occlusion lies at the heart of the clinical burden imposed by SCD. More than just a molecular event, it is the gateway to a cascade of debilitating complications that define the day-to-day reality of individuals living with this inherited blood disorder. The term “vaso-occlusion” refers to the obstruction of blood flow within the microvasculature, primarily due to the abnormal adhesion of sRBCs, leukocytes, and platelets to the endothelial lining of blood vessels. This occlusion results in tissue ischemia, acute pain, organ dysfunction, and cumulative damage that progressively worsens the patient’s quality of life and life expectancy[43]. One of the most striking and immediate clinical manifestations of vaso-occlusion is the vaso-occlusive crisis (VOC) – a hallmark feature of SCD. These episodes are characterized by sudden and severe pain, often in the bones, joints, chest, or abdomen, and are the leading cause of emergency hospital visits and admissions among SCD patients. Painful crises may last from hours to several days, and their frequency and intensity vary widely between individuals. Recurrent VOCs not only disrupt normal life but also lead to chronic pain syndromes that are difficult to manage and often require long-term opioid therapy[44]. Beyond pain, vaso-occlusion is directly implicated in several organ-specific morbidities. For instance, in the lungs, repeated VOCs can culminate in acute chest syndrome (ACS) – a potentially fatal complication marked by chest pain, fever, hypoxia, and pulmonary infiltrates. In the brain, vaso-occlusion underlies the development of ischemic strokes, particularly in children, making SCD one of the leading causes of pediatric stroke. Silent cerebral infarcts, which occur without overt symptoms, are even more common and are associated with cognitive deficits and poor academic performance[45].

The kidneys are also highly vulnerable. Repeated vaso-occlusion in renal microvasculature leads to sickle cell nephropathy, manifesting as impaired urine concentrating ability, proteinuria, and ultimately chronic kidney disease. Similarly, bone infarction due to impaired blood flow can lead to avascular necrosis, especially of the femoral and humeral heads, significantly impairing mobility. Splenic infarction due to vaso-occlusion begins early in life, leading to functional asplenia by the age of 5 in most individuals with homozygous SCD. This loss of splenic function contributes to immunodeficiency and a heightened risk of life-threatening infections. Vaso-occlusion also plays a significant role in the chronic hemolytic anemia seen in SCD. Trapped and damaged RBCs are prematurely destroyed in the microvasculature, leading to chronic anemia, fatigue, and compensatory cardiac stress[43]. The cumulative impact of these vaso-occlusive events significantly drives mortality in SCD. While improved healthcare and early interventions have increased survival, many individuals still succumb prematurely due to complications stemming from repeated vascular occlusion. Acute events, such as ACS, stroke, and sepsis, often rooted in or exacerbated by vaso-occlusion, remain leading causes of death. Furthermore, the chronic end-organ damage resulting from years of recurrent microvascular insults contributes to progressive multi-organ failure in adulthood. In essence, vaso-occlusion is not merely a pathological feature – it is the epicenter of suffering in SCD. It connects the molecular defect in hemoglobin to the broad spectrum of clinical complications that burden patients^[44,45]^.

### Pathways to mitigate vaso-occlusion in SCD

Vaso-occlusion in SCD is a critical pathological event that leads to pain, organ damage, and decreased quality of life. It occurs when sRBCs aggregate, obstructing blood flow through the microvasculature. This blockage is often exacerbated by the interaction of sickle cells with other blood components, such as leukocytes, platelets, and endothelial cells, mediated by adhesion molecules. In recent years, a deeper understanding of the molecular mechanisms behind vaso-occlusion has led to the development of several promising therapeutic strategies. These aim not only to alleviate symptoms but also to target the underlying processes that drive these obstructions. Below are several key pathways to mitigate vaso-occlusion in SCD^[43,44]^.

#### Targeting adhesion molecules

Adhesion molecules are central to the pathogenesis of vaso-occlusion in SCD. These molecules, such as selectins, integrins, and VCAM-1, mediate the interaction between sRBCs, leukocytes, platelets, and the endothelial cells lining the blood vessels. By blocking these interactions, it is possible to reduce the incidence of microvascular occlusions. Monoclonal antibodies targeting adhesion molecules such as **E-selectin** (e.g., Uproleselan) and **P-selectin** have shown promising results in clinical trials, reducing the frequency of vaso-occlusive crises. Integrin inhibitors, such as **Natalizumab**, have also been tested for their ability to prevent the firm adhesion of sickle cells to endothelial surfaces. These therapies aim to interrupt the early stages of the adhesion cascade, thus preventing the aggregation of cells that leads to vascular blockage^[45,46]^.

#### Modulating RBC deformability

The sickling of RBCs leads to their reduced deformability, making it difficult for these cells to pass through small blood vessels. This contributes directly to the mechanical blockage of capillaries and arterioles. One pathway to mitigate vaso-occlusion is through the enhancement of RBC deformability. Drugs, like **hydroxyurea**, which increase the production of fetal hemoglobin (HbF) have been shown to reduce sickling by decreasing the polymerization of HbS. This reduces the rigidity of RBCs, helping them maintain their ability to flow through microvasculature. Additionally, novel therapies that directly target RBC membrane properties or promote red cell hydration, such as **Voxelotor**, aim to reduce the extent of sickling and improve blood flow in affected tissues^[47,48]^.

#### Anti-inflammatory strategies

Inflammation plays a pivotal role in promoting adhesion molecule expression on endothelial cells and leukocytes, further contributing to vaso-occlusion. Inflammatory cytokines such as TNF-α, IL-1, and IL-6 upregulate the expression of adhesion molecules, leading to increased leukocyte and sRBC adhesion. Anti-inflammatory therapies, such as **hydroxyurea** and **JAK inhibitors**, aim to decrease the inflammatory response by inhibiting cytokine release or blocking downstream signaling pathways. **Hydroxyurea**, in particular, has become a cornerstone of SCD management, not only for its ability to reduce sickling but also for its anti-inflammatory effects. More targeted approaches, such as **anti-TNF therapy**, could potentially reduce the endothelial activation and leukocyte adhesion that contribute to vaso-occlusion in SCD[49].

#### Enhancing nitric oxide (NO) bioavailability

Nitric oxide (NO) is a critical molecule in maintaining vascular health. It promotes vasodilation, reduces platelet aggregation, and inhibits the adhesion of sickle cells to the endothelium. In SCD, NO bioavailability is often reduced due to oxidative stress, which inactivates NO and promotes vasoconstriction. Enhancing NO bioavailability could therefore be an effective strategy to mitigate vaso-occlusion. Therapies such as **L-arginine supplementation**, which is a precursor of NO, and **nitric oxide donors** are being explored to restore the NO levels in the vasculature. Additionally, **hydroxyurea** has been shown to increase NO production, contributing to its beneficial effects on vaso-occlusion. By promoting vasodilation and reducing endothelial cell activation, these therapies can potentially reduce the severity of vaso-occlusive events^[50,51]^.

#### Gene therapy and gene editing approaches

Gene therapy offers a groundbreaking approach to modifying the genetic underpinnings of SCD. The most well-known strategy involves the insertion of a modified **β-globin gene** to produce hemoglobin F (HbF) to alleviate sickling and its associated complications. Gene editing techniques like **CRISPR-Cas9** have also been used to directly correct the mutations in the β-globin gene or to induce the expression of fetal hemoglobin, reducing the prevalence of sickled cells in circulation. Additionally, gene editing could be used to modify endothelial cells to reduce the expression of adhesion molecules, thus reducing the likelihood of sickle cell adhesion and vaso-occlusion. Although gene therapy is still in the early stages of clinical application, it holds significant promise as a potential long-term solution for mitigating vaso-occlusion in SCD^[52,53]^.

#### Blood transfusions

Blood transfusions are a well-established therapy for patients with severe SCD, particularly in those with frequent or severe vaso-occlusive episodes. By increasing the proportion of normal RBCs in circulation, blood transfusions can reduce the number of sickled cells, thereby improving blood flow and reducing the risk of occlusion. Exchange transfusions, in particular, are used to rapidly decrease the percentage of sRBCs in the body. While blood transfusions are effective in preventing acute vaso-occlusive events, their long-term use is associated with complications such as iron overload, which requires chelation therapy. Despite these challenges, blood transfusions remain a vital component of therapy, especially for high-risk patients^[54,55]^.

#### Platelet inhibition

Platelets contribute to vaso-occlusion by promoting the formation of microthrombi in response to endothelial injury and sickle cell adhesion. Inhibition of platelet activation may help reduce the formation of these occlusive thrombi. Drugs, such as **aspirin,** and newer antiplatelet agents are being investigated for their potential to reduce platelet aggregation in SCD. While the benefits of platelet inhibition in SCD remain under study, early results suggest that reducing platelet-mediated thrombus formation could help reduce the frequency of vaso-occlusive events. Further research is needed to determine the optimal use of antiplatelet therapies in this context^[56,57]^.

#### Combination therapies

Given the multifactorial nature of vaso-occlusion in SCD, combination therapies that target different aspects of the pathophysiology may be more effective than single-agent treatments. For instance, combining hydroxyurea with selectin inhibitors or NO donors could offer complementary benefits, addressing both the deformability of RBCs and the adhesion properties that contribute to vaso-occlusion. Additionally, combining gene therapy with existing treatments, such as blood transfusions or hydroxyurea, may provide a more comprehensive approach to managing the disease. As research continues, the development of combination regimens tailored to the specific needs of individual patients could provide the best outcomes in preventing vaso-occlusive crises^[58,59]^.

### Therapeutic approaches targeting adhesion molecules in SCD

SCD is a complex, chronic condition that is primarily characterized by repeated vaso-occlusive episodes. These episodes are driven by interactions between sRBCs, leukocytes, platelets, and endothelial cells, all of which are mediated by adhesion molecules. These molecules facilitate the sticking of sickled cells to the endothelial lining, leading to blood flow obstruction and subsequent organ damage. Given their central role in the pathophysiology of vaso-occlusion, adhesion molecules have emerged as key targets for therapeutic intervention in SCD. Various strategies aimed at blocking or modulating these adhesion molecules hold promise for reducing the frequency and severity of vaso-occlusive crises and improving the clinical outcomes for patients with SCD^[60,61]^.

### Monoclonal antibodies targeting selectins

One of the most promising approaches to targeting adhesion molecules in SCD involves the use of monoclonal antibodies that block selectins, such as E-selectin and P-selectin. Selectins are cell surface glycoproteins that mediate the initial tethering and rolling of leukocytes and sickle cells on the endothelial surface, a critical step in the formation of microvascular occlusions. By blocking these selectins, it is possible to prevent the adhesion of these cells to the endothelium, thereby reducing the likelihood of vaso-occlusion. Several monoclonal antibodies targeting selectins have been investigated in preclinical and clinical studies. For instance, **Uproleselan (GMI-1271)**, an E-selectin antagonist, has shown promise in clinical trials by reducing the frequency of vaso-occlusive events and improving blood flow in SCD patients. This approach aims to disrupt the early stages of the adhesion cascade and thus prevent the formation of obstructive blood clumps in microvessels^[62,63]^.

## Integrin blockade

Integrins are another class of adhesion molecules that play a crucial role in the firm adhesion of cells to the endothelium. Integrins such as α4β1 (VLA-4) interact with VCAM-1 on endothelial cells, mediating the adhesion of sRBCs and leukocytes. Targeting these integrins offers another therapeutic strategy to reduce vaso-occlusion. **Natalizumab**, a monoclonal antibody that targets α4β1 integrins, is used clinically for other conditions, such as multiple sclerosis and Crohn’s disease, and has been tested in SCD. Preclinical studies suggest that integrin blockade can significantly reduce the adhesion of sickle cells and neutrophils to the endothelium, mitigating the formation of blood clots in small vessels. However, despite promising results, the clinical use of integrin inhibitors in SCD requires careful consideration of potential side effects, such as increased risk of infections and other immune-related complications^[64,65]^.

### Blocking P-selectin and platelet adhesion

P-selectin is another key adhesion molecule that facilitates the interaction between platelets, leukocytes, and the endothelial cells, thereby contributing to the formation of microthrombi during vaso-occlusion. By blocking P-selectin, it may be possible to reduce platelet aggregation and the subsequent inflammatory cascade that exacerbates microvascular obstruction. Several therapeutic agents are being developed to target P-selectin, including monoclonal antibodies such as **Rucaparib**. Early-phase clinical trials suggest that blocking P-selectin can reduce platelet aggregation and inflammation in SCD, although further studies are needed to assess its clinical efficacy. Moreover, these therapies may also have benefits for other inflammatory conditions beyond SCD, thereby expanding their therapeutic potential^[66–77]^.

### Gene therapy and gene editing approaches

In addition to directly targeting adhesion molecules with pharmacological agents, gene therapy and gene editing strategies offer innovative approaches for modulating the adhesion molecules involved in SCD pathogenesis. One such approach is to modify the expression of adhesion molecules on the surface of sRBCs or endothelial cells. For example, the genetic editing of sickle cell hemoglobin (HbS) using CRISPR-Cas9 technology holds the potential to not only correct the underlying genetic mutation but also to reduce the adhesion properties of sickle cells. In addition, gene therapy could target endothelial cells to decrease the expression of adhesion molecules like E-selectin, VCAM-1, and ICAM-1, thereby preventing the recruitment and adhesion of inflammatory cells to the vessel walls. While gene therapy remains an emerging field, recent advancements show promise in editing genes that could modulate the adhesion mechanisms central to vaso-occlusion in SCD^[68,69]^.

### Inhibitors of inflammatory pathways

Inflammation is a critical driver of adhesion molecule expression in SCD. As a result, targeting inflammatory pathways that upregulate the expression of adhesion molecules is another strategy for preventing vaso-occlusion. For example, **hydroxyurea**, a well-established therapy for SCD, works in part by reducing the levels of pro-inflammatory cytokines and adhesion molecules. The drug has been shown to increase fetal hemoglobin (HbF) production, reduce leukocyte count, and decrease the expression of adhesion molecules like E-selectin. Newer therapies, such as **Janus kinase (JAK) inhibitors**, which block inflammatory signaling pathways like the JAK-STAT pathway, are being explored as potential adjuncts to hydroxyurea therapy. These agents may further reduce inflammation, endothelial activation, and the expression of adhesion molecules, thus contributing to the reduction of vaso-occlusion in SCD^[70–72]^.

### Combination therapies

Given the complexity of the pathophysiology of SCD, combination therapies that target multiple points in the adhesion cascade may be more effective than monotherapies. For example, combining selectin inhibitors with integrin antagonists or anti-inflammatory agents could provide a more comprehensive approach to managing vaso-occlusion. Such combinations could simultaneously prevent the initial adhesion of sickle cells, reduce platelet aggregation, and mitigate the inflammatory responses that exacerbate microvascular obstruction. Clinical studies investigating the safety and efficacy of combination therapies are ongoing, and early results suggest that these approaches may provide more robust benefits in reducing vaso-occlusive crises and improving patient outcomes^[73,74]^.

## Conclusion

Adhesion molecules play a central role in the pathophysiology of vaso-occlusion in SCD, driving the interactions between sRBCs, leukocytes, platelets, and endothelial cells that lead to microvascular blockages, inflammation, and organ damage. Targeting these adhesion molecules presents a promising therapeutic strategy to mitigate the severity and frequency of vaso-occlusive crises, which are the hallmark of SCD. Therapeutic approaches such as monoclonal antibodies targeting selectins, integrins, and P-selectin, as well as gene therapy and anti-inflammatory interventions, have shown potential in preclinical and clinical trials, demonstrating their ability to reduce cellular adhesion, inflammation, and microvascular obstruction.

## Data Availability

Not applicable as this is a narrative review.

## Research registration unique identifying number (UIN)

Not applicable as this is a narrative review.
